# Very High Density of Chinese Hamster Ovary Cells in Perfusion by Alternating Tangential Flow or Tangential Flow Filtration in WAVE Bioreactor™—Part II: Applications for Antibody Production and Cryopreservation

**DOI:** 10.1002/btpr.1703

**Published:** 2013-05-21

**Authors:** Marie-Françoise Clincke, Carin Mölleryd, Puneeth K Samani, Eva Lindskog, Eric Fäldt, Kieron Walsh, Véronique Chotteau

**Affiliations:** 1 School of Biotechnology, Cell Technology Group, KTH (Royal Institute of Technology)Stockholm, SE-10691, Sweden; 2 GE Healthcare Bio-Sciences AB, Björkgatan 30SE-75184 Uppsala, Sweden; 3 GE Healthcare Bio-Sciences CorpWestborough MA, 01581

**Keywords:** wave bioreactor, ATF, TFF, ultrafiltration, comparison between perfusion and fed-batch, MAb production, cryopreservation

## Abstract

A high cell density perfusion process of monoclonal antibody (MAb) producing *Chinese* hamster ovary (CHO) cells was developed in disposable WAVE Bioreactor™ using external hollow fiber (HF) filter as cell separation device. Tangential flow filtration (TFF) and alternating tangential flow (ATF) systems were compared and process applications of high cell density perfusion were studied here: MAb production and cryopreservation. Operations by perfusion using microfiltration (MF) or ultrafiltration (UF) with ATF or TFF and by fed-batch were compared. Cell densities higher than 10^8^ cells/mL were obtained using UF TFF or UF ATF. The cells produced comparable amounts of MAb in perfusion by ATF or TFF, MF or UF. MAbs were partially retained by the MF using ATF or TFF but more severely using TFF. Consequently, MAbs were lost when cell broth was discarded from the bioreactor in the daily bleeds. The MAb cell-specific productivity was comparable at cell densities up to 1.3 × 10^8^ cells/mL in perfusion and was comparable or lower in fed-batch. After 12 days, six times more MAbs were harvested using perfusion by ATF or TFF with MF or UF, compared to fed-batch and 28× more in a 1-month perfusion at 10^8^ cells/mL density. Pumping at a recirculation rate up to 2.75 L/min did not damage the cells with the present TFF settings with HF short circuited. Cell cryopreservation at 0.5 × 10^8^ and 10^8^ cells/mL was performed using cells from a perfusion run at 10^8^ cells/mL density. Cell resuscitation was very successful, showing that this system was a reliable process for cell bank manufacturing. © 2013 American Institute of Chemical Engineers *Biotechnol. Prog*., 29:768–777, 2013

## Introduction

Fed-batch mode is widely used for the production of glycoprotein.^1^ This mode is typically based on the use of concentrated feeds and stoichiometric delivery of needed components in particular amino acids.^2^,^3^ Often, a strategy of minimizing the by-product production is used.^4^,^5^ Interestingly, simulation models have shown that the cost of goods per gram of produced antibody is the same for perfusion and fed-batch.^6^,^7^ Lim et al.^6^ pointed out that perfusion was more favorable economically owing to a lower initial investment but that looking at the failure and contamination risk fed-batch was preferred. Systematic and experimental comparisons of production by fed-batch and perfusion are difficult to perform and can be irrelevant owing to the influence of many parameters such as the cell-specific productivity, the optimization level of the fed-batch, the perfusion length, the scale, and so forth. However, they can be used for some benchmarking: 6-, 7-, and 1.4-fold higher production in perfusion than in fed-batch for HeLa, SF-9 insect, and Chinese hamster ovary (CHO) cells, respectively, has been reported.^8^–^10^ Fed-batch process can result in higher level of protease, lower cell viability, and higher accumulation of lactate and ammonium, whereas perfusion provides a constant environment more favorable for the health of the cells and for the quality of the product of interest. This is particularly true when a cell-specific perfusion rate is applied.^11^,^12^

Cell separation by filtration has an inherent drawback, which is filter fouling. It was described for spin filter and crossflow filtration that fouling is caused by deposition of nucleic acid and dead cells.^13^,^14^ Filter fouling is accompanied by the retention of the product of interest inside the bioreactor. Hiller et al.^15^ installed a back-flush pump to prevent this phenomenon as they observed that up to 20% of produced antibody were retained by the hollow fiber (HF) filter cartridge in their hybridoma perfusion process. Back-flushing is systematically obtained by alternating tangential flow (ATF) system, which is described to prevent fouling^16^; however, this effect has not been quantified.

The concept of continuous filtration to concentrate high-molecular-weight components, that is retaining the product of interest in the bioreactor and eliminating low-molecular-weight components (e.g., toxic by-products, lactate, and ammonium) was introduced in continuous dialysis bioreactor.^17^–^19^ The same principle is now used in industry using ultrafiltration (UF) HF perfusion, that is XD Process Technology at DSM, achieving very high cell densities^20^; however, experimental details have not been disclosed. The advantage of this process is to retain the product of interest inside the bioreactor so that harvesting of the bioreactor is performed once instead of weekly/bi-weekly harvests of the perfused permeate. This process cannot be as long as conventional perfusion owing to the risk of degradation of the product of interest or even the risk of aggregation from high-protein concentration.

Cell cryopreservation in amounts large enough to directly inoculate a bioreactor has been described some years ago for hybridoma cells.^21^ Cryopreservation in bags is widely used for blood cell storage and has been studied for suspension of CHO and BHK cells stored from shake flask or bioreactor culture at 20 × 10^6^ cells/mL^22^,^23^ or transient expression of HEK293 cells in suspension.^24^ Over the last decade, cell banking of large cell amounts, achieved by high cell density and large stored volume, has had an increasing interest in the biopharmaceutical industry. It has the obvious advantage of a gain of 2–3 weeks of time and labor compared to the classical approach of small-volume cryovials at low cell density.

This article, performed with an IgG1 producing CHO cell line, reports the results obtained together with Part I.^25^ The cell growth and associated observation of broth viscosity using microfiltration (MF) perfusion were presented in Part I for two perfusion runs using MF tangential flow filtration (TFF), TFF#6 and TFF#10, and four runs using MF ATF, ATF#5, ATF#8, ATF#9, and ATF#15A-B. Part II of this study focuses on the applications of perfusion by HF: the monoclonal antibody (MAb) production using MF ATF or MF TFF, the production obtained using the same perfusion settings with an external hollow fiber ultrafilter cartridge (UF HF), a comparison with fed-batch production, and cell bank manufacturing from high-cell-density bioreactor.

## Materials and Methods

### Perfusion culture processes

The experimental procedure for MF perfusion was described in Part I. The same procedure was applied for UF perfusion except for the following. The cells were continuously circulated through the lumen side of the UF HF, using a flow rate of 1.5 L/min (shear rate, 2,790 s^−1^). In ATF and TFF systems, the UF HFs were RTPUFP-50-C-5A (GE Healthcare) with 50 kDa pore size, 0.5 mm lumen, 520 polysulfone fibers, 30 cm nominal flow path length, and 2,000 cm^2^ filter area.

### Fed-batch cultures in Cellbag™

The cells were inoculated in the same Cellbag™ as in perfusion process at a seeding cell density of 0.8 × 10^6^ cells/mL in 2 L animal component-free IS CHO CD XP medium with hydrolysate (Irvine Scientific), supplemented with 2 mM glutamine (Irvine Scientific) and Streptomycin/Penicillin G/Amphotericin B (SAFC) as in perfusion process. The feeding strategy consisted in adding 225, 150, and 75 mL feed medium, 15/15%/% Efficient Feed A/B (Invitrogen) in IS CHO CD XP medium, on days 1–7, 8, and 9-end, respectively. Glucose and glutamine boost additions were performed daily according to the cell need. The set points were 35% DO, pH 7, and 37°C temperature or 37°C decreased to 35.5°C on day 7. The pH and DO were controlled as in perfusion process. The rocking rate, angle, and gas flow rate were 20–22 rpm, 5–6°, and 0.1 L/min with O_2_ concentration up to 30%.

### Computational equations of the kinetics rates

For the fed-batch process, the apparent cell growth rate (*µ*), the cell-specific rates of glutamine consumption (*q*_gln_), and ammonium production (*q*_amm_) were calculated as follows:


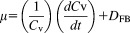
(1)


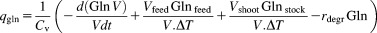
(2)


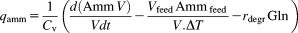
(3)

where *C*_v_ *=* cell density, *V* = bioreactor volume, *D*_FB_ = dilution rate *dV/(V dt)*, Gln = glutamine concentration, Amm = ammonium concentration in the culture; subscripts feed, stock, and shoot refer to daily added feed medium, stock solution concentration used for shoots and daily added shot volume of stock solution; *ΔT* = time interval between additions (*ΔT =*1 day); *r*_degr_ = degradation rate of glutamine, measured to be 0.046 day^−1^ in IS CHO CD XP medium at 37°C by recording the degradation during 7 days. Similar equations were used for the cell-specific rates of glucose consumption (*q*_glc_), lactate production (*q*_lac_), and MAb production (*q*_MAb_) except that the degradation term was skipped.

For the perfusion process, the variable computation was presented in Part I. The MAb cell-specific productivity and the accumulated harvested MAb production (HT_tot_) were calculated as follows:


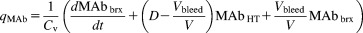
(4)


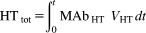
(5)

with MAb_brx_ = MAb concentration in the bioreactor, MAb_HT_ = MAb concentration in the harvest line, *D =* perfusion rate, *V*_bleed_ = bleed volume, *V*_HT_ = harvest volume in perfusion or fed-batch runs.

### Cryopreservation and cell thaw tests

Cells were frozen either at 10^8^ or at 0.5 × 10^8^ cell in 1 mL vial by taking the cell broth directly from the bioreactor: the cell broth was collected with a syringe and was put immediately on ice. It was then either proceeded as such or diluted at 1:2 with fresh Ex-Cell 302 (SAFC) (Ex-Cell 302 medium was selected for this operation for two reasons: the cells could pass from IS CHO CD XP medium to Ex-Cell 302, and vice versa, without adaptation; and Ex-Cell 302 ACF medium had been used in the research cell bank and cell expansion in shake flask for historical reasons). In both cases, 10% dimethyl sulfoxide (DMSO) (SAFC) was added before vial storage at −80°C in insulated box for 1 day, followed by liquid N_2_ storage. The time between collecting the cell broth from the bioreactor and the storage at −80°C was ≤5 min. The cell thaw was performed in shake flasks at 10^6^ cells/mL: a cryo-vial was thawed in 37°C water bath until <20% of iced cell could still be observed; the cells were then transferred in a shake flask and prewarmed Ex-Cell 302 with 4 mM glutamine (named “medium A” here) was added. DMSO was eliminated on day 1 by centrifugation (5 min, 100*g*) and resuspension in fresh medium A at 10^6^ cells/mL. Afterward, the cells were passaged in medium A as described in Part I. The productivity test consisted in prolonging the batch cultivation in shake flask and measuring the MAb production.

## Results and Discussion

### Perfusion with UF HF

Two runs were performed to evaluate the perfused bioreactor using UF either mounted with ATF (ATF#18) or mounted with TFF (TFF#21). The same settings as MF HF perfusion were used except the following items: (1) the HFs were 50 kDa UF with 2,000 cm^2^ filter area instead of MF, (2) the recirculation flow rate was increased compared to MF HF owing to the larger filter area. The recirculation rate was the maximal flow rate possible of the ATF-2, that is 1.5 L/min. This flow resulted in shear rate of 2,790 s^−1^, whereas 1 L/min in MF HF resulted in shear rate of 3,400 s^−1^ owing to MF's smaller fiber number, 50 instead of 520. Although the shear rate was lower than using MF, the recirculation rate was higher and hence a preliminary study to deem the pumping deleterious effect on the cells was performed (see the next paragraphs).

**Figure 1:**
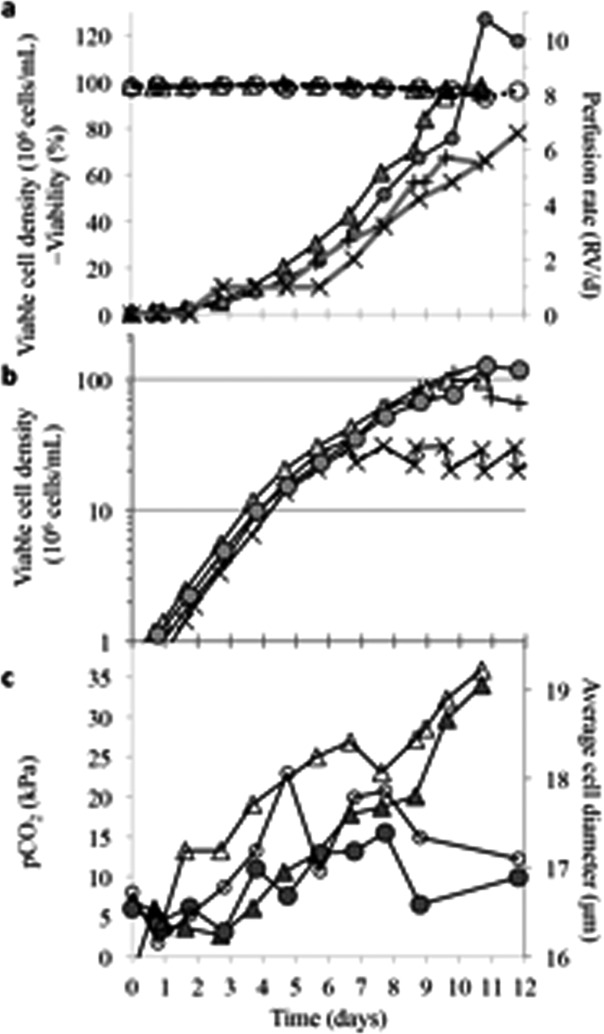
(a) Perfusion processes using UF ATF, run ATF#18 (triangle) or UF TFF, run TFF#21 (circle): viable cell density (plain symbols), viability (open symbols), and perfusion rate: ATF#18 (+), TFF#21 (x); (b) logarithmic viable cell density of runs ATF#18 and TFF#21 in comparison with MF HF perfusion in ATF#15A-B (+) and TFF#10 (x); (c) pCO2 (plain symbols) and average cell diameters (open symbols) for ATF#18 (triangle) and TFF#21 (circle) runs.

After an exponential growth comparable in both systems, maximal cell densities of 1.27 × 10^8^ and 1.01 × 10^8^ cells/mL were reached in TFF#21 and ATF#18 runs, respectively, after 11 and 12 days (Figure 1a). The growth rates were comparable to those observed using MF HF with a fast exponential growth until ∼30 × 10^6^ cells/mL followed by a slower rate (Figure 1b). On day 12, run TFF#21 suffered from erroneous control settings, leading to too high DO (∼100%) and a subsequent viability drop to 88% (data following day 12 not shown). In ATF#18 run on day 10, pCO_2_ reached 30 kPa, a growth inhibitory level,^26^ (Figure 1c), and hence the rocking rate and angle were increased from 22 to 26 rpm, 7° to 23–27 rpm, 7.2°, to improve CO_2_ removal; a higher agitation being avoided to prevent air bubble occurrence in the dip tube (Part I). Increasing the agitation, and thus the gas transfer, did not prevent pCO_2_ from further increasing from 30 to 34 kPa on day 11, (Figure 1c), causing interruption of cell growth. Furthermore, on day 11, the PRV exhaust decreased to −0.75 bar (or −11 psi) and the ATF motion stopped a few hours later owing to insufficient vacuum similar to that was observed using MF ATF (Part I). During the TFF perfusion, the pCO_2_ was kept under 16 kPa even at high cell density as the rocking rate and angle could be increased with limited risk of damage from air occurrence in the dip tube. This pCO_2_ level was comparable to the runs using MF HF at similar cell densities (Part I). In ATF#18 run after day 9, at cell density ≥0.8 × 10^8^ cells/mL, the average cell diameter (ACD) significantly increased, reaching 19.2 µm on day 11 (Figure 1c). A larger cell diameter is typical for cell growth arrest. On the contrary, using the TFF system, the ACD was constant (around 17.5 µm) after day 4.

#### Pressure in UF HF

**Figure 2:**
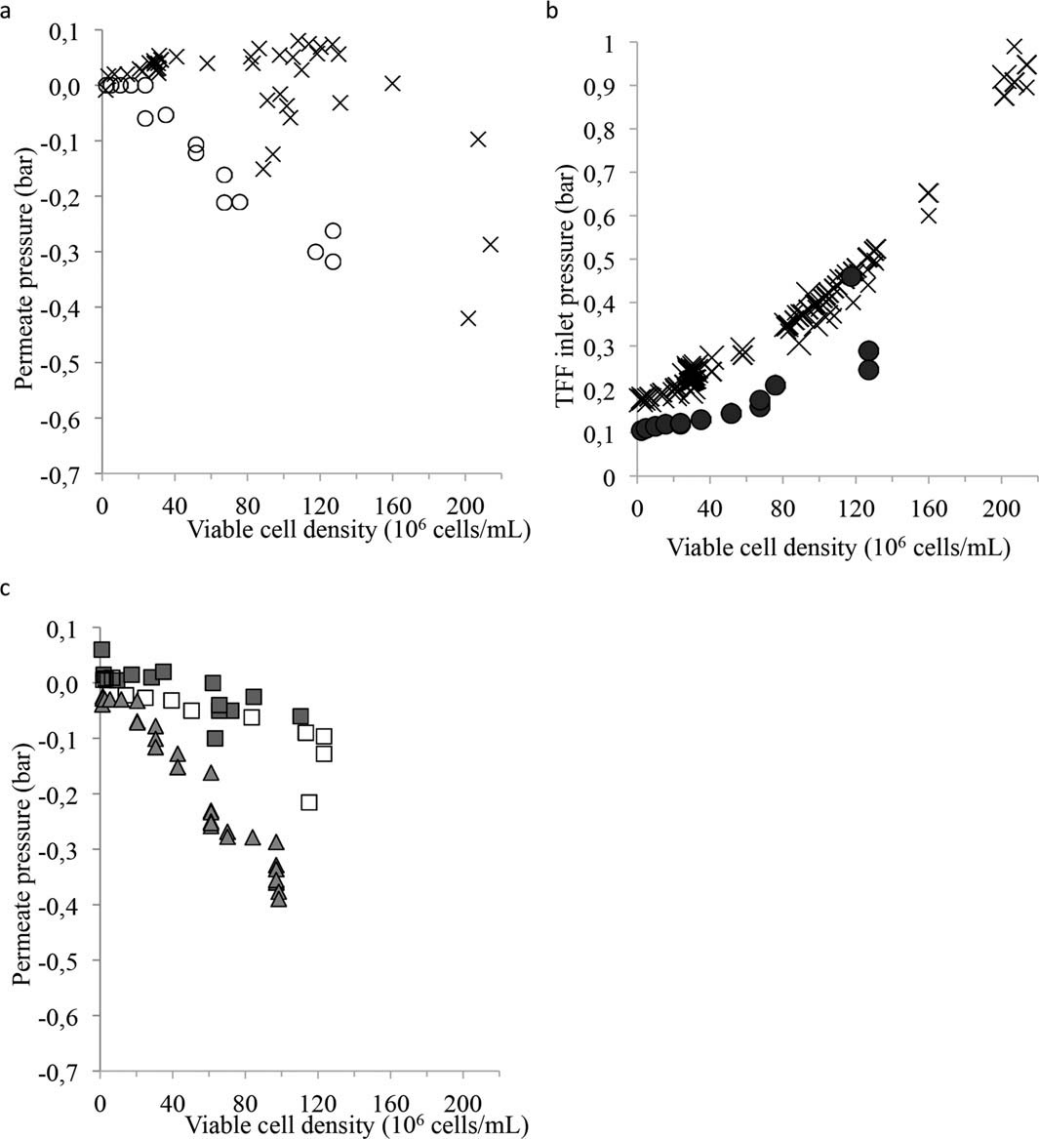
(a) Permeate pressure during UF TFF run, TFF#21 (circle) and MF TFF run TFF#10 (x) run; (b) inlet pressure during UF TFF run, TFF#21 (circle) and MF TFF run, TFF#10 (x); (c) permeate pressure during UF ATF run, ATF#18 (triangle) and MF ATF run, ATF#15A (plain squares) and ATF#15B (open square).

Contrary to the cultures using MF membrane, the permeate pressure (*P*_2_) in UF ATF run (ATF#18) and UF TFF run (TFF#21) decreased linearly with increasing cell density and perfusion rate for cell densities of >20 × 10^6^ cells/mL (Figures 2a,c). *P*_2_ reached −0.3 to −0.4 bar at 10^8^ cells/mL density operated at *D* = 5.5 RV/day. As expected, the pore size of the UF HF required stronger harvest suction than the MF HF. As shown in Figure 2b, using UF HF in TFF#21 run the inlet pressure (*P*_1_) in function of the cell density was lower but had a trend comparable to the one obtained using MF HF in TFF#10 run. As observed in Part I, a high correlation could also be observed between *P*_1_ and the cell broth viscosity, however, with a smaller proportional factor owing to the different filter, resulting in a different hydrodynamic resistance. As mentioned above, on day 12 at 1.17 × 10^8^ cells/mL density, the viability dropped owing to elevated DO; simultaneously *P*_1_ increased to 0.47 bar. It continued to increase up to 0.8 bar the following 2 days, whereas the viability dropped to 82% and the cells did not grow anymore (data not shown). The increased *P*_1_ can probably be attributed to the low cell viability creating cell debris and DNA residue; but not to the high cell density as it had been higher, 1.27 × 10^8^ cells/mL on day 11, whereas *P*_1_ was lower (0.26 bar).

### Effect of recirculation pump on cells

In the TFF system, shear stress can be obtained from the passage in the HF or in the tubing system, for example edges, connections, and from the recirculation pump. An experiment was performed to quantify this latter effect by pumping the cell broth in the same settings as used in the TFF system but with the HF short circuited. Different recirculation rates up to 2.75 L/min were applied every day, showing no impact on the cell growth and viability, which remained ≥95% (Table 1).

**Table 1:** Effect on the Cell Growth and Viability of Different Recirculation Speeds by Pumping Using a Watson Marlow 620S (Two Rollers)

Pump Speed (L/min)	1.5	1.8	2	2.25	2.5	2.75
Viable cell density (10^6^ cells/mL) at initial time	3.5	2.1	2.4	1.8	1.8	1.8
Viable cell density (10^6^ cells/mL) (after time [hours])	4.5 (24)	4 (24)	4.2 (24)	2.6 (15)	4.2 (48)	7.1 (72)
Viability (%) at initial time	96.8	94.5	95.9	95.2	95.2	95.2
Viability (%) after 24 h	96.2	95.9	96.8	95.1	97.8	97.1

### Fed-batch cultures

**Figure 3:**
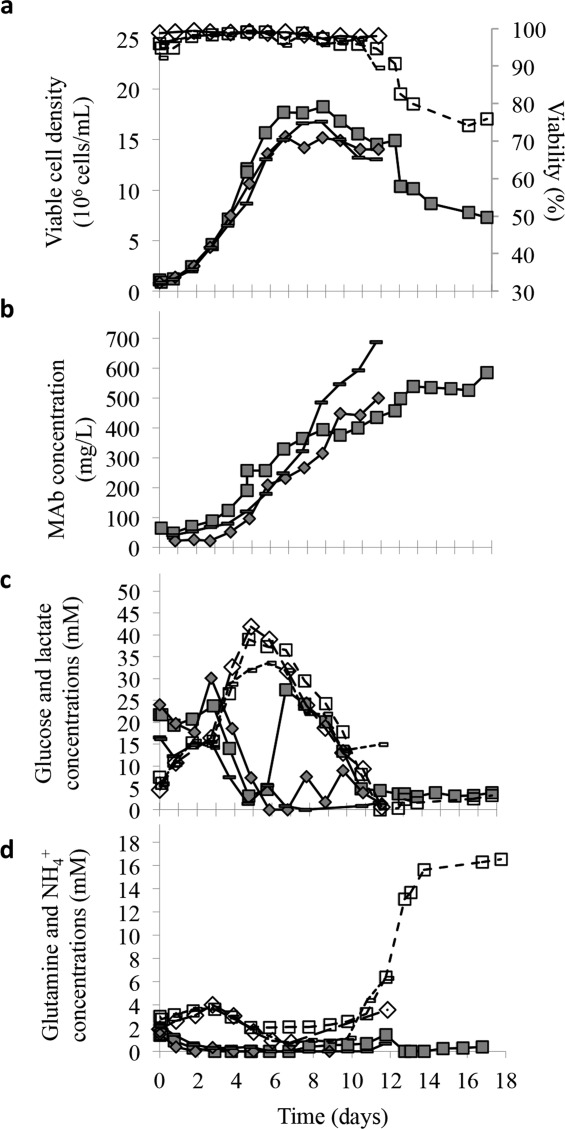
(a) Viable cell density (continuous lines) and viability (dotted lines) in duplicate fed-batch runs FB#11 (square), FB#22 (dash), and in fed-batch run FB#16 (diamond) with identical settings except for temperature decrease to 35.5°C applied at day 7; (b) concentrations of MAb; (c) concentrations of glucose (continuous) and lactate (dotted) in the bioreactor; (d) concentrations of glutamine (continuous) and ammonium (dotted) in the bioreactor.

**Figure 4:**
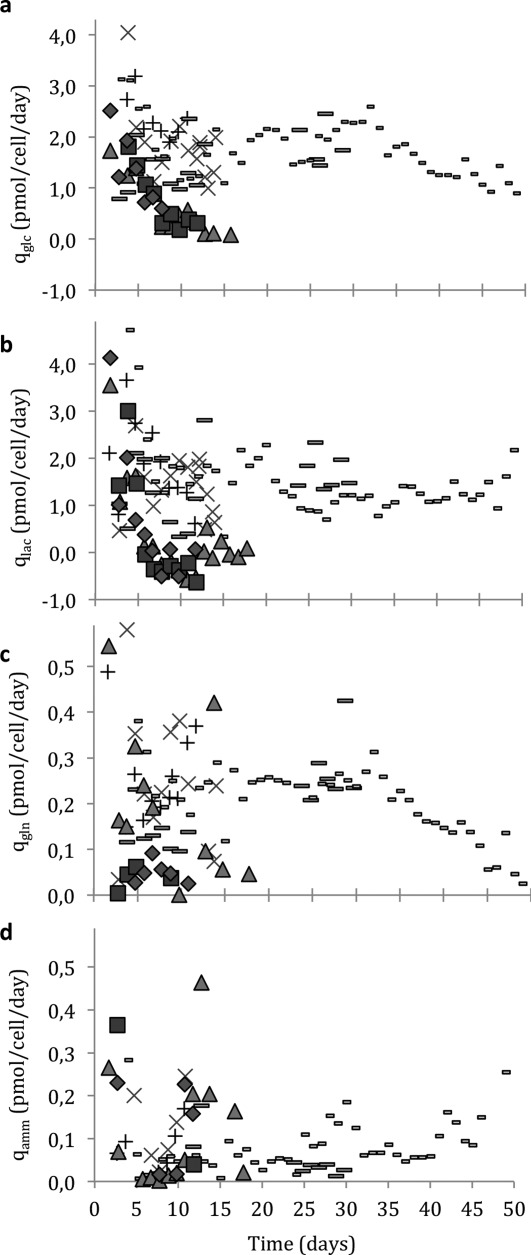
(a) Cell-specific consumption of glucose in perfusion runs using UF ATF, ATF#18 (+), UF TFF TFF#21 (x), MF ATF ATF#15 (long dash), MF TFF#10 (short dash), and in fed-batch runs FB#11 (triangle), FB#16 (square), and FB#21 (diamond); (b) cell-specific production of lactate; (c) cell-specific consumption of glutamine; (d) cell-specific production of ammonium.

Three runs, FB#11, FB#16, and FB#22, were performed in fed-batch mode for a comparison with perfusion in the identical bioreactor. The fed-batch runs were identical except FB#16 run, which had a temperature shift (**Materials and Methods** section). In all the fed-batch runs, the cells had a growth with a maximal *µ* = 0.7 d^−1^, an exponential growth until days 6–7 and a maximal viable cell density of 15–18 × 10^6^ cells/mL (Figure 3a). This cell density pattern was typical for a fed-batch culture as described in studies reported in previously published literature.^3^,^27^ The viability was high (≥95%) until day 11 and the mild hypothermia at 35.5°C improved the viability (≥97%). The concentrations of MAb, glucose, glutamine, lactate, and ammonium, (Figures 3b–d) were typical for a fed-batch process with MAb accumulating over time, low glucose and glutamine concentrations (except glucose from day 7 in FB#11 owing to manipulation error), low final lactate concentration and high final ammonium concentration.^28^–^30^ The specific rates *q*_glc_ and *q*_lac_ were high at the beginning of the fed-batch cultures and progressively decreased with reduced cell growth as often reported for fed-batch process (Figures 4a,b).^31^,^32^ The *q*_gln_ and *q*_amm_ data were noisier but it was observed that after day 10 *q*_amm_ increased (Figures 4c,d). In comparison, these rates were comparable between day 5 and the run-end during all the perfusion runs using MF or UF, ATF or TFF, except for higher *q*_gln_ and lower *q*_amm_ at cell density of >1.6 × 10^8^ cells/mL (Part I).

### MAb production in UF ATF and UF TFF perfusion runs

**Figure 5:**
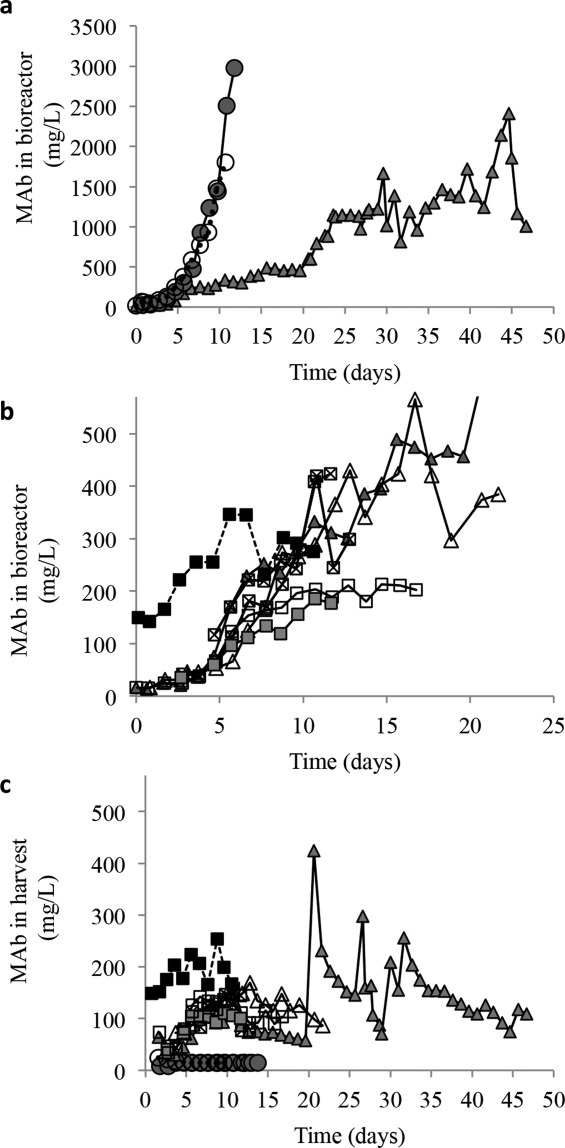
(a) MAb concentrations in bioreactor in UF ATF#18 (open circle), UF TFF#21 (plain circle), and MF TFF#10 (plain triangle); (b) MAb concentrations in bioreactor in MF runs ATF#15 (crossed square), ATF#9 (black square), ATF#8 (plain square), ATF#5 (gray square), and TFF#6 (open triangle) (for other legends, see (a)); (c) MAb concentrations in harvest line (for other legends, see (b)).

The MAb concentration in the bioreactor (MAb_brx_) increased rapidly to 1,801 mg/L in ATF#18 (on day 11) and 2,978 mg/L in TFF#21 (on day 12) and at similar rate, whereas it stayed at the lower detection limit in the harvest line (Figures 5a,c). A fast MAb accumulation was expected owing to the increasing cell density and the 50-kDa membrane completely retaining the MAbs in the bioreactor.

### MAb production in MF ATF and MF TFF perfusion runs

A comparable pattern was observed in all the MF perfusion runs except run ATF#8 and run TFF#10 after day 20: MAb_brx_ and the MAb concentration in the harvest line (MAb_HT_) increased rapidly during the first 6–7 days, then MAb_HT_ stabilized around 93–158 mg/L, whereas MAb_brx_ stabilized around 176–243 mg/L on day 9–10, thus at a higher level than MAb_HT_ and with a 3-day delay (Figures 5a–c). A slow MAb_HT_ decrease and MAb_brx_ increase with time were even observed in some runs, for example TFF#10 on days 10–20. Run ATF#8, inoculated at 5.5 × 10^6^ instead of 0.45 × 10^6^ cells/mL, generated a faster MAb production with comparable MAb_HT_ profile shifted 3 days upward. Higher MAb_brx_ and MAb_HT_ were observed in ATF#8 and TFF#6 runs, probably owing to the transitory use of another medium (Materials and Methods in Part I) and to *D* = 1.3 RV/day instead of 1.5 RV/day in the other runs. This higher production was confirmed by computing the volumetric production (data not shown), and *q*_MAb_ (see below). Notice that MAb_brx_ above 400 mg/L on day 11 in ATF#15A-B was owing to the interruption of ATF function (Part I) and should not be taken into account here.

MAb_HT_ and MAb_brx_ stabilization is typical for perfusion run at different cell densities using constant CSPR,^11^,^12^ as applied here: a larger MAb amount produced by a higher cell density is more diluted when using a higher perfusion rate. In case of complete MAb transfer from the bioreactor to the harvest, MAb_HT_ and MAb_brx_ are identical; however, they were different here owing to a partial MAb retention by the HF. Furthermore, the concurrent slow MAb_brx_ increase and MAb_HT_ decrease were owing to a MAb retention increasing with time, for example in TFF#10 run, the 1^st^ HF lasted 30 days, during which time filter fouling increased.

Interestingly, a transitory period of higher MAb_HT_ was observed immediately subsequent to a significant increase of the perfusion rate on day 21 in TFF#10 run. This was followed by a transitory down slope. It is probable that the sudden change of the flow rate through the MF HF pores temporarily removed the cake fouling the membrane; however, it reformed after a few days as can be seen from MAb_HT_ decrease. This observation is in agreement with the use of back flush to prevent fouling as in the ATF operation^16^ or HF TFF back-flush pumping.^15^ After day 20, MAb_HT_ was ≍154 mg/L and MAb_brx_ became ≥1,000 mg/L with a slight continuous increase with time, indicating an important MAb retention. MAb_HT_ profile showed several peaks, which were approximately mirrored by reversed variations in MAb_brx_, probably subsequently to transitory property changes of the fouled HF. On day 41 and onward, the perfusion rate was increased again. However, this time, MAb_HT_ stayed stable, whereas MAb_brx_ increased highly and correlated with the cell density increase. At this stage, the HF was probably irreversibly fouled so that the cake could not be removed as observed day 21. In agreement with this, irreversible cake formation of this HF was confirmed 8 days later, that is after run completion, following the method described by Russotti et al.^33^ (data not shown).

### MAb Harvest

**Figure 6:**
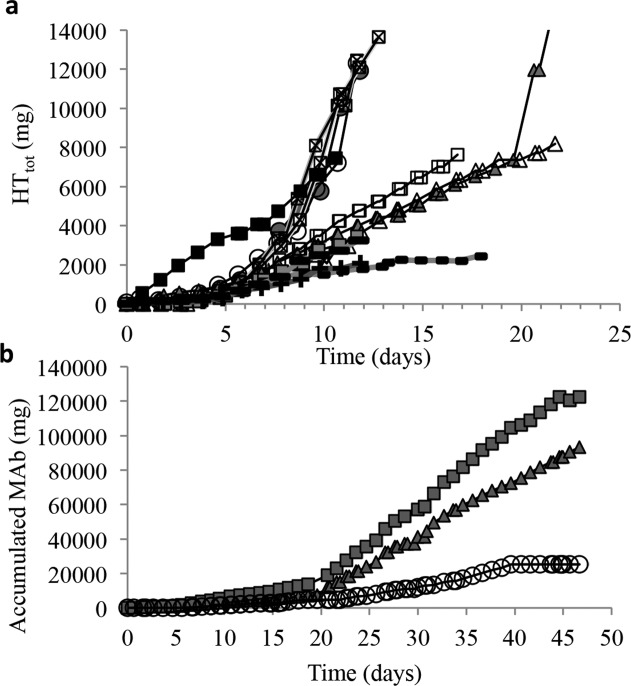
(a) Accumulated harvested MAb production (for legend, see Figures 5a,b). HT tot in run MF TFF#10 (plain triangle), only partly represented in (a) can be seen completely in (b); (b) representation of HT_tot_ (plain triangle) in run MF TFF#10, the total accumulated cellular production (square), sum of the production in the harvest, the production loss in the discarded cell bleeds and the residual amount in the bioreactor, and the accumulated MAb amount lost in the discarded cell bleeds (circle).

The accumulated harvested MAb production, one of the most important parameters for the process, is shown in Figure 6a. In the MF runs stabilized at ∼25 × 10^6^ cells/mL cell density (ATF#5, ATF#8, ATF#9, TFF#6, and TFF#10 until day 20), the slope of HT_tot_ was comparable. This was also true for ATF#8 run in which MAb accumulated earlier owing to the higher initial cell density. Higher cell densities resulted in steeper slope (ATF#15A-B or TFF#10 from day 20). In Figure 6b, HT_tot_ in run TFF#10 is shown together with the total accumulated MAb amount really produced by the cells, MAb_tot_


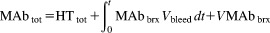
(6)

MAb_tot_ was higher than HT_tot_ by a difference, sum of the MAb loss in the cell bleeds and another smaller loss from the residual MAb left in the bioreactor at the run completion. An illustration is summarized in Table 2 where MAb loss in bleeds and residual in bioreactor are presented for ATF #9 and TFF#10 runs for more than 17 days with a cell density maintained at 25 × 10^6^ cells/mL.

**Table 2:** MAb Loss in ATF#9 and TFF#10 for More Than 17 days With Cell Densities Maintained at 25 × 10^6^ cells/mL

Run	Harvest HT_tot_	Loss of MAb in Bleeds	Loss of residual MAb in Bioreactor	Cellular Production MAb_tot_
TFF#10	6,073 mg	3,771 mg	1,896 mg	11,740 mg
	52%	32%	16%	100%
ATF#9	7,628 mg	2,311 mg	764 mg	10,702 mg
	71%	22%	7%	100%

#### Analysis of the Yields

**Figure 7:**
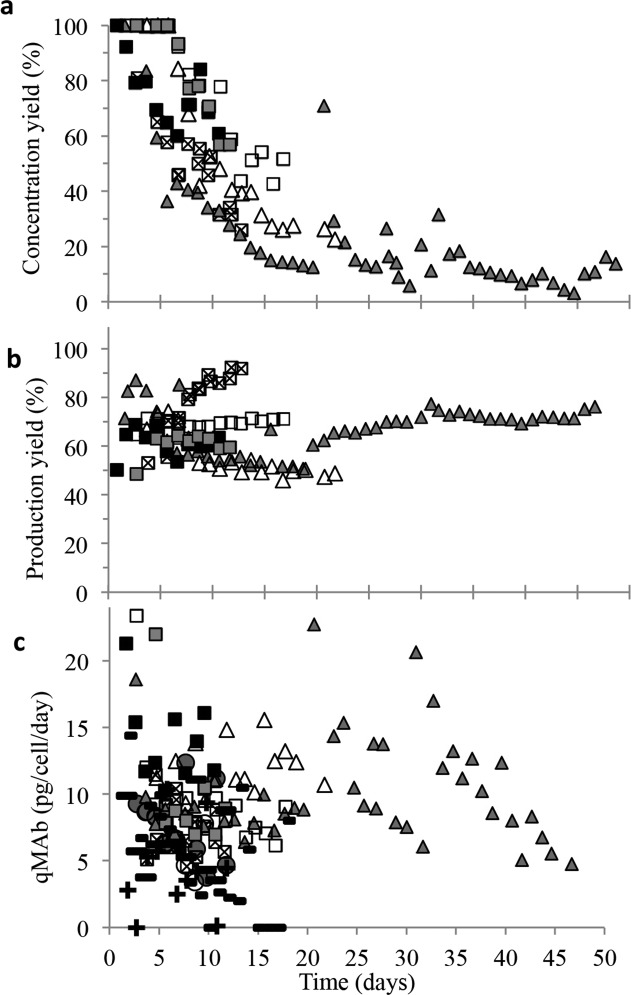
(a) Concentration yield, *Y*_conc_, ratio of the concentrations in the harvest line and in the bioreactor (for legend, see Figures 5a,b); (b) production yield, *Y*_prod_, ratio of the accumulated harvested MAb production and the total accumulated cellular production; (c) cell-specific MAb production in perfusion runs, for legend, see Figures 5a,b, and fed-batch runs FB#11 (short dash), FB#16 (+), FB#22 (long dash).

The concentration yield (*Y*_conc_), ratio MAb_HT_/MAb_brx_, was 100% during the first days and then decreased rapidly with time, reflecting the partial retention (Figure 7a). After the first days, the production yield (*Y*_prod_), ratio HT_tot_/MAb_tot_, became significantly higher than *Y*_conc_ as expected owing to its integral character: for instance, in MF ATF#15A-B performed without bleeds, *Y*_conc_ decreased to 50% or less after 10 days, whereas *Y*_prod_ increased to 86–92% (Figure 7b). In the runs stabilized at 25 × 10^6^ cells/mL density, *Y*_prod_ was between 54 and 71% and between 46 and 56% when using ATF and TFF, respectively, after the first days. It was concluded that there was a MAb retention using both systems but using TFF caused a larger retention and hence the ATF system was more favorable for the MAb production than the TFF system. This has been described theoretically^16^; however, to our knowledge, this is the first time that data are published. The yield difference was higher in the case of production at stable cell density with cell bleeds as larger amounts of MAbs were discarded. Another drawback of MAb retention by the HF is an increase of the MAb residence time inside the bioreactor, which is, of course, an issue for sensitive glycoproteins.^12^

*Y*_conc_ decreased with time and with increasing cell density, probably owing to a higher amount of cell debris and nucleic acid in a similar way as reported for the filter fouling in spin filter.^13^ The debris and nucleic acids were not eliminated from the bioreactor except in the cell bleeds.

#### Effect of Shear Rate

Recirculation rates of 0.3–1 L/min, that is shear rate of 1,000–3,400 s^−1^, were applied (Table 1 in Part I). Increasing the recirculation rate did not improved *Y*_conc_, for example TFF#10 run had slightly lower yield than TFF#6 run on days 9–19 for recirculation rates 1 and 0.3 L/min, respectively (Figure 7b). These shear rates were probably not high enough to observe a favorable effect from increasing the shear rate as in yeast MF harvest for shear rates of ≥8,740 s^−1^.^33^ However, a higher recirculation rate was more favorable for ATF function, that is for reduced air occurrence in the dip tube (Part I).

#### Comparison of MAb production in perfusion using MF or UF HF and fed-batch

Figure 6a shows HT_tot_ for the different runs performed at different cell densities and in the absence or presence of bleeds. Runs UF ATF#18, UF TFF#21, and MF ATF#15A-B resulted in comparable HT_tot_. As discussed above, the cell bleeds caused a lower production for MF TFF compared to MF ATF. The MAb production obtained in the fed-batch runs was at the lower edge of the perfusion runs. MAb harvested after 12 days in UF and MF, ATF, and TFF perfusion runs as well as fed-batch were compared based on data in the absence of cell bleed for UF ATF#18, UF TFF#21, MF ATF#15B, MF TFF#10 (for which a calculation was made based on the real data) and average of FB#11, FB#16, and FB#22: the harvested MAb production was comparable and ∼12 g for all the perfusion runs and was 2 g in fed-batch cultures, that is six times lower than in perfusion. Yuk et al.^34^ reported a sevenfold higher titer of recombinant oncolytic adenoviral vector in perfusion using ATF compared to fed-batch in an infection process of HeLa. Jardin et al.^10^ obtained a production of Secreted Alkaline Phosphatase eightfold higher in perfusion using acoustic settler than in fed-batch mode in an SF-9-BEV insect cell culture. Meuwly et al.^9^ converted a CHO perfusion process into fed-batch. The productivity per bioreactor unit volume and year was ≍70% in fed-batch compared to perfusion in packed-bed bioreactor.

Illustrating the potential MAb production in the perfusion system used here, in run TFF#10, a total of 93 g of MAb was harvested after 47 days although this run was a study run with periods at cell density maintained at 25 × 10^6^ cells/mL and 1.1 × 10^8^ cells /mL before reaching 2 × 10^8^ cells/mL. Furthermore, it was calculated from the data of this run that 34 g could be harvested over a 17-day period of culture including cell expansion followed by cell density maintained at 10^8^ cells/mL and that 22 g was harvested per week while maintaining the cell density at 10^8^ cells/mL. In other words, a 1-month culture would generate 57 g MAb in this 4 L working volume process using this model cell line with moderate cell-specific productivity in view of today's industrial standard. The comparison of perfusion and fed-batch is, of course, very difficult to make as it depends on the cell density and the perfusion culture length but other factors such as the scalability and the total cost of goods covering upstream and downstream processes should also be taken into account.^6^

*q*_MAb_ was comparable in all the ATF and TFF runs, ∼9 pg/cell/day, except ATF#8 and TFF#6 runs partly performed with another medium, ∼13 pg/cell/day (Figure 7c). It also seems that *q*_MAb_ decreased somewhat in TFF#10 run from day 42 at a density of ≥1.31 × 10^8^ cells/mL. This could potentially be attributed to the high cell density as the cell line was stable as shown by a productivity test performed from cells frozen at 2 × 10^8^ cells/mL on day 44 (data not shown). *q*_MAb_ in fed-batch was in average comparable or lower than in the perfusion runs.

### Cell banking and seed expansion

**Figure 8:**
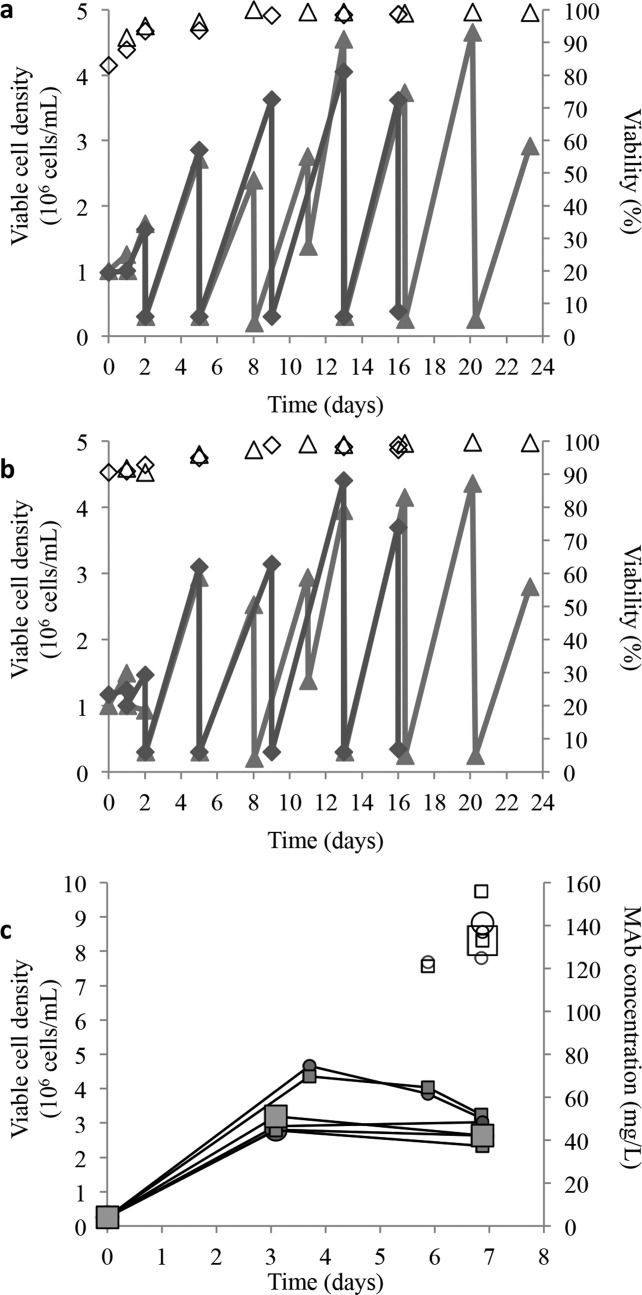
Cell thaw (duplicate) from (a) 108 and (b) 0.5 × 10^8^ cells/mL vials cryopreserved from 10^8^ cells/mL culture ATF#15B day 12; (c) duplicate productivity test performed from one of the cell thaw cultures presented in (a) (circle) and (b) (square) at passages 7 and 8: viable cell density (plain symbols) and MAb concentration (open symbols); and productivity test performed at passage 8 with supplementary addition of 200 nM methotrexate (large symbols).

Cells taken from ATF#15B run at 10^8^ cells/mL cell density on day 12 (i.e., day 30 of the whole ATF#15A-B run) were cryopreserved in 1-mL vials. The cells were frozen at cell concentrations of 10^8^ and 0.5 × 10^8^ cells/mL, either by adding 10% DMSO to the cell broth or by diluting with fresh medium before adding 10% DMSO. The cell resuscitation and subsequent growth were excellent with viability in average 90–91% on day 1 and higher after this day and cell growth already observed from day 1 or 2 (Figures 8a,b). Duplicate productivity tests were performed from one of the cell thaw cultures at passages 7 and 8 as well as a productivity test at passage 8 with supplementary addition of 200 nM methotrexate (MTX) (Figure 8c). After 1 week, average MAb concentrations and cell-specific MAb productivity of 138 mg/L and 7.1 pg/cell/day, respectively, were reached as typical for productivity test with this cell line. No effect of MTX addition was observed, indicating as well that the cells were stable and that no cell subpopulation insensitive to MTX had emerged during the 30 days of ATF#15A-B total culture period.

This study clearly demonstrates that CHO cell cryopreservation from a perfusion culture at high cell densities around 10^8^ cells/mL is feasible, allowing high cell number with high cell growth and viability at thaw. Ninomiya et al.^21^ reported high cell density freezing at 1.5 × 10^8^ cells/mL for hybridoma cells in the presence of 20% serum, whereas this study was performed without serum. Tao et al.^35^ developed a CHO cell bank manufacturing process in which the cells were expanded up to a cell density of 27 × 10^6^ cells/mL in a wave-induced bioreactor perfused with an interior filter separation device. They increased the final cell density of their cell bank at 0.9–1 × 10^8^ cells/mL by centrifugation. It is probable that a controlled bioreactor environment instead of shake flask expansion is more beneficial for the cell health, the reproducibility, and reliability of the cryopreservation process.

## Conclusions

Applications of high-cell-density perfusion process of MAb producing CHO cells in disposable WAVE Bioreactor™ using TFF or ATF were studied here using MF or UF. Cell densities higher than 10^8^ cells/mL were obtained using UF TFF or UF ATF. Comparable amounts of MAb were produced by the cells in perfusion by ATF or TFF, MF or UF; however, the MAbs were partially retained by the MF. The retention was higher using the TFF than the ATF system. The main MAb loss caused by this retention was product discarded from the bioreactor in the daily bleeds to maintain a constant cell density. The MF TFF was thus less favorable for the production of MAb in comparison with MF ATF. From this point of view, the UF TFF and UF ATF had a major advantage compared with the MF HF as these were aimed at retaining completely the product. They also allowed a reduction of the MAb dilution, harvest from the bioreactor culture, 4 L, instead of from the accumulated harvests (≍100 L in the present cases). As expected, the perfusion process generated a higher MAb production than the fed-batch by a factor of 6 after 12 days and a factor of 28 in a 1-month perfusion at a constant cell density of 10^8^ cells/mL. Together with Part I, our study showed that perfusion up to 1.3 × 10^8^ cells/mL had no influence on the cell metabolism and MAb production when applying a constant cell-specific perfusion rate. The equipment, that is the WAVE Bioreactor™, the ATF and the TFF, was robust. The operational experience from this study indicated no difference concerning the HF capacity using ATF or TFF. Recirculation rates of 1 L/min (shear rate, 3,400 s^−1^) using MF HF and 1.5 L/min (2,790 s^−1^) using UF HF were well tolerated by CHO cells using ATF or TFF systems. The effect of pumping by the TFF system in the absence of HF was studied. It indicated that the pumping used in the present perfusion setting had no detrimental effect on the cells. Cell cryopreservation from high-cell-density bioreactor culture was very successful. This system was more reliable and robust for cell bank manufacturing than the classical approach of shake flask expansion as the cells were manufactured in a controlled bioreactor in the presence of a stable environment generated by the perfusion.
